# Two layers of fascia envelop the pudendal nerve canal: a cadaver study

**DOI:** 10.1007/s00384-026-05114-w

**Published:** 2026-03-03

**Authors:** Kenro Chikazawa, Satoru Muro, Tomoyuki Kuwata, Keiichi Akita

**Affiliations:** 1 Department of Obstetrics and Gynecology, Saitama Medical Center, Jichi Medical University, 1-847, Amanuma-Cho, Omiya-Ku, Saitama 330-8503 Japan; 2 Department of Clinical Anatomy, Institute of Science Tokyo, 1-5-45 Yushima, Bunkyo City, Tokyo 113-8510 Japan

**Keywords:** Pelvic anatomy, Alcock’s canal, Fascial planes, Pudendal nerve, Cadaver study

## Abstract

**Purpose:**

This study aimed to examine the pudendal nerve in the context of tumors involving the pudendal canal and to clarify its anatomical course and the structural composition of Alcock’s canal, while elucidating the relationships among the obturator internus nerve, sacrotuberous ligament, and fascia of the obturator internus muscle.

**Methods:**

Six cadavers (12 pelvic halves) were dissected. The dissections focused on the pudendal canal, particularly the positions and interrelationships of the fasciae, muscles, ligaments, and surrounding fascial structures from medial and posterior perspectives.

**Results:**

The sacrotuberous ligament comprised two distinct layers, with the pudendal canal located within its structure. The proper fascia was distinguishable and situated on the muscle side of the obturator internus muscle. The pudendal nerve was traced within the fascia of the sacrotuberous ligament, whereas the obturator internus nerve coursed between the obturator internus muscle and its proper fascia. Coronal section examination of the right pelvis confirmed that the pudendal nerve was enveloped by fascia. The obturator internus nerve ran along the muscle side of the obturator internus fascia, occupying a layer distinct from that of the pudendal nerve.

**Conclusions:**

The pudendal nerve travels within the fascia derived from the sacrotuberous ligament and does not pass through the fascia of the obturator internus. The proper fascia of the obturator internus muscle is located relatively close to the muscle, and the obturator internus nerve courses between the obturator internus muscle and its own fascia. These findings describe the anatomy of the pudendal canal and may provide a foundation for future surgical investigation.

**Supplementary Information:**

The online version contains supplementary material available at 10.1007/s00384-026-05114-w.

## Introduction

There is an increasing number of studies on the resection of recurrent tumors involving the pudendal canal, particularly those employing laterally extended endopelvic resection in pelvic surgery [1, 2]. Long-term prognostic data emphasize the importance of precise anatomical understanding in surgical planning for tumors involving the pudendal canal. However, surgical techniques vary, with some studies recommending resection of the sacrotuberous ligament, sacrospinous ligament, and ischial spine [1, 3]. These variations in the surgical approach are likely attributable to the limited number of anatomical studies on this subject available to surgeons.

Traditional anatomical literature has often described muscles and ligaments in isolation, offering limited insights into their interrelationships and the surgical planes critical to oncological outcomes [4]. Furthermore, few detailed studies by clinicians described the continuity of tissues and fascial layers, which are essential for successful tumor resection in the evolving field of pelvic oncology. Anatomical studies have shown that the sacrotuberous ligament connects the sacrum to the ischial tuberosity and extends medially as a falciform ligament toward the ischial tuberosity [5, 6]. Therefore, further anatomical investigation of the pudendal canal may help guide surgical strategies for recurrent tumor resection in this region.

The aim of this study was to examine the pudendal nerve, which is often resected with tumors infiltrating the pudendal canal, by clarifying its anatomical course and the structural composition of Alcock’s canal. We also sought to elucidate the spatial relationships between the obturator internus nerve, the sacrotuberous ligament, and the fascia of the obturator internus muscle. Accordingly, the present study is a descriptive anatomical investigation designed to clarify these structural relationships.

## Methods

### Preparation of the cadaveric specimens

Cadavers donated to our department were used in accordance with Japanese law and the Act on Body Donation for Medical and Dental Education (Act Number 56 of 1983). Prior to death, all donors voluntarily provided consent for their bodies to be used for educational and research purposes. The study methods were explained in detail, and informed consent was obtained from all donors. Following death, consent was reconfirmed with the donors’ families to ensure that there were no objections.

The cadavers were preserved by arterial perfusion with 8% formalin and subsequently stored in 30% alcohol. This study was approved by the local ethics committee (approval number M2018-006). All procedures were conducted in accordance with relevant ethical guidelines and regulations.

### Macroscopic anatomy

Macroscopic examinations were performed on the cadavers. Ten pelvic halves were dissected to examine the pudendal canal, with particular attention to the positional and spatial relationships among the fasciae, muscles, ligaments, and surrounding fascial structures from medial and posterior perspectives.

### Histology

Histological analysis was performed on one cadaver. Tissue samples were collected from three distinct sites along the course of the pudendal nerve within the fascia of the obturator internus muscle using a diamond band pathology saw (EXAKT 312; EXAKT Advanced Technologies, Norderstedt, Germany). The samples were fixed in 10% formalin, decalcified using Plank–Rychlo solution (126.7 g/L AlCl_3_·6H_2_O, 85 mL/L HCl, 50 mL/L HCOOH), and dehydrated. The tissues were subsequently embedded in paraffin, sectioned into 5-μm-thick slices, and stained with Masson’s trichrome stain.

The stained slides were scanned using a high-resolution scanner (GT-X980; EPSON, Suwa, Japan), and detailed images were obtained using a digital slide scanner (NanoZoomer-SQ C13140; Hamamatsu Photonics, Hamamatsu, Japan).

## Results

Six cadavers (12 pelvic halves; mean age at death, 84.3 years; age range, 66–97 years) were used in this study. The sacrotuberous ligament was incised at its medial extension toward the ischial tuberosity to allow detailed observation of the pudendal canal. As the course of the pudendal nerve was traced, the surrounding fascial structures became clearer, and we observed that the pudendal nerve canal and the pathway of the obturator internus nerve were anatomically distinct. Specifically, the sacrotuberous ligament consisted of two distinct layers that contained the pudendal canal within its structure. Additionally, the proper fascia of the obturator internus muscle was identified as a separate fascial layer located on the muscle side of the obturator internus.

**Fig. 1:**
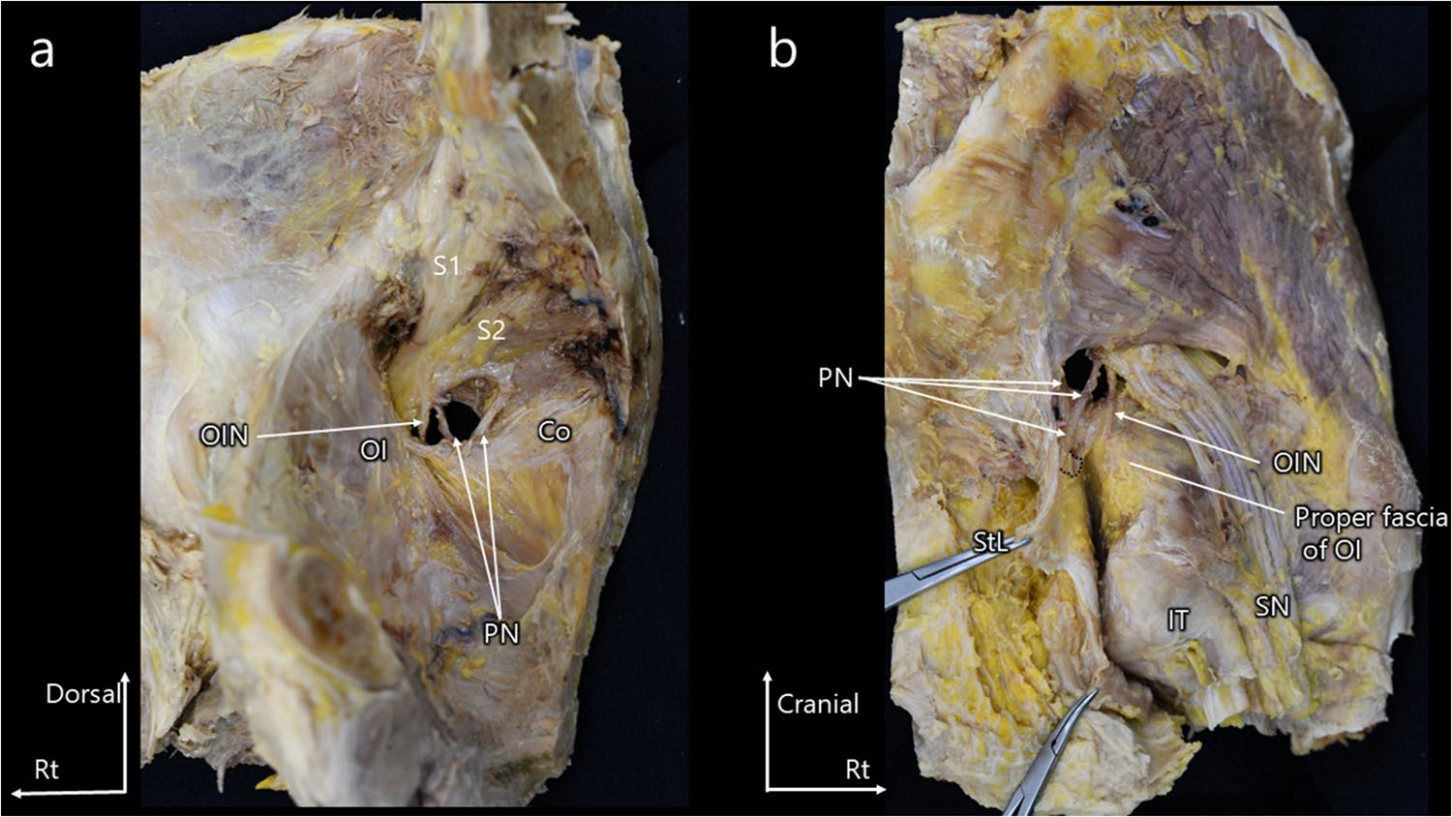
(**a**) Medial view of the pelvis. (**b**) Lateral view of the pelvis. Anatomical course of the pudendal and obturator internus nerves in the pelvis. The pudendal and obturator internus nerves are visualized on the medial and lateral sides of the pelvis. Tracing the pudendal nerve reveals that it runs within the fascia of the sacrotuberous ligament, whereas the obturator internus nerve courses between the obturator internus muscle and its proper fascia. Co, coccygeus muscle; IT, ischial tuberosity; OI, obturator internus muscle; OIN, obturator internus nerve; PN, pudendal nerve; SN, sciatic nerve; StL, sacrotuberous ligament

The pudendal and obturator internus nerves were visualized on the medial and lateral sides of the pelvis (Fig. 1a, b). The pudendal nerve was traced and found to run within the fascia of the sacrotuberous ligament, whereas the obturator internus nerve coursed between the obturator internus muscle and its proper fascia.

**Fig. 2:**
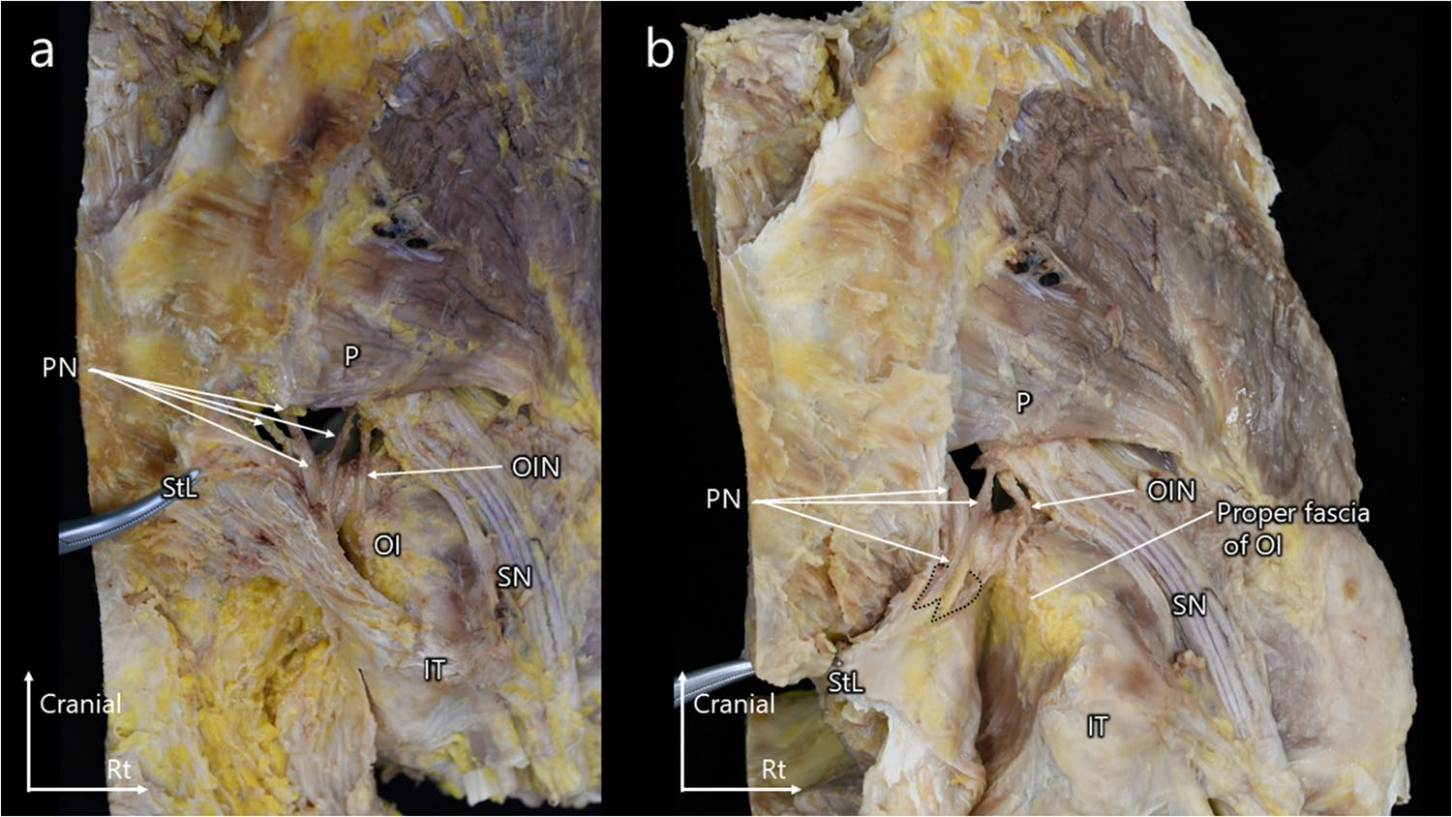
(**a**) Lateral view of the pelvis after incision of the sacrotuberous ligament. (**b**) Lateral view after further reflection of the sacrotuberous ligament, exposing the obturator internus muscle and its proper fascia. Relationship of the sacrotuberous ligament with the pudendal canal, obturator internus muscle, and obturator internus nerve. The sacrotuberous ligament consists of two distinct layers forming the pudendal canal, and the proper fascia of the obturator internus muscle is a separate structure situated on the muscle side. The pudendal nerve continues to follow the fascia of the sacrotuberous ligament, whereas the obturator internus nerve remains aligned with the fascia of the obturator internus muscle. Co, coccygeus muscle; IT, ischial tuberosity; OI, obturator internus muscle; OIN, obturator internus nerve; P, piriformis muscle; PN, pudendal nerve; SN, sciatic nerve; StL, sacrotuberous ligament

To improve visualization, the sacrotuberous ligament was further incised proximally. This confirmed that the sacrotuberous ligament comprises two distinct layers forming the pudendal canal and that the proper fascia of the obturator internus is a discrete structure situated on the muscle side. The pudendal nerve continued to follow the fascia of the sacrotuberous ligament, whereas the obturator internus nerve remained aligned with the fascia of the obturator internus muscle (Fig. 2a, b). Removal of adipose tissue from the ischioanal fossa and dissection of the fasciae of the sacrotuberous ligament and the obturator internus muscle further clarified these anatomical relationships (Online Resource 1).

**Fig. 3:**
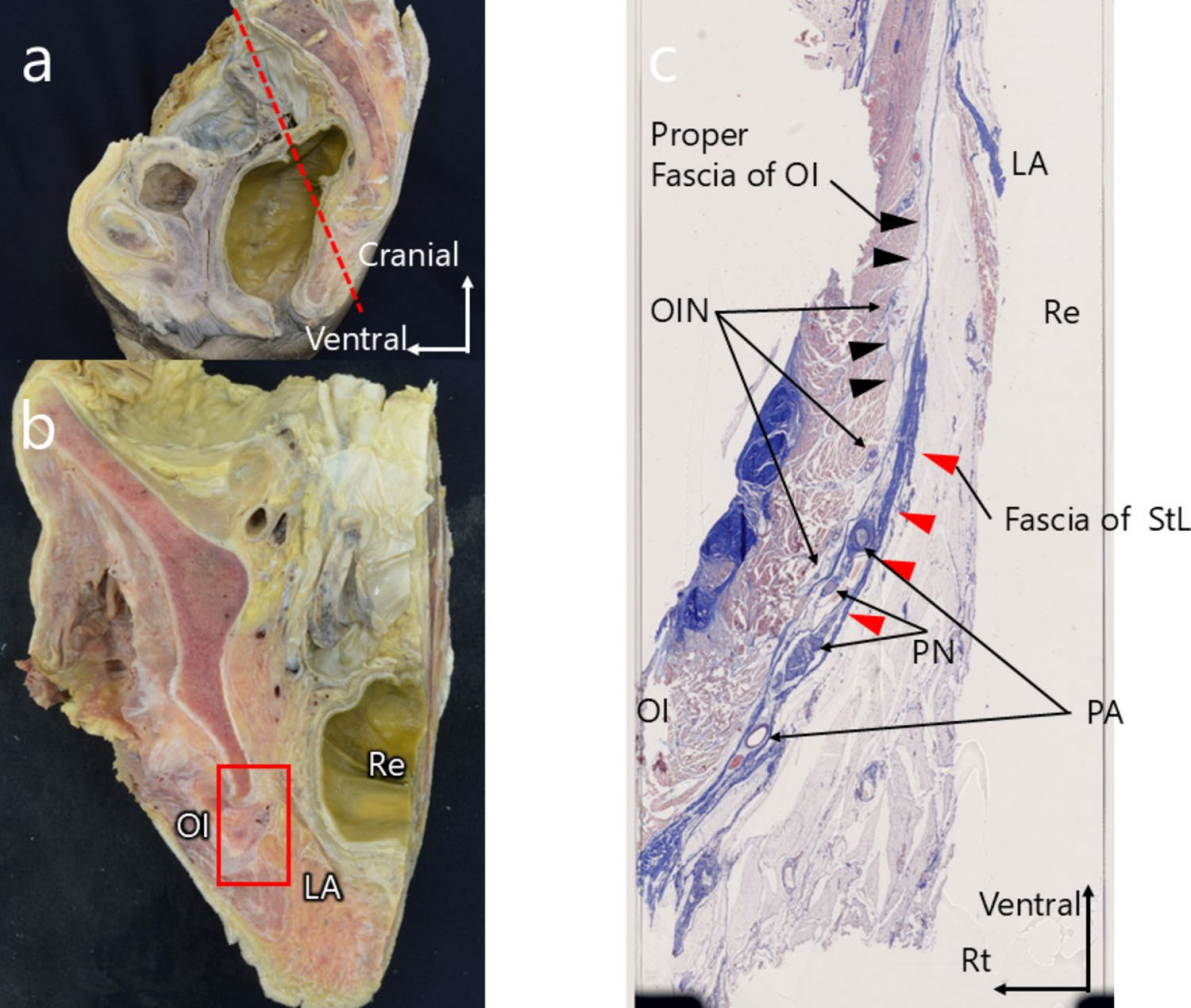
(**a**) Sagittal section of the pelvis. (**b**) Coronal section through Alcock’s canal indicating the sampling plane for histological examination. (**c**) Histological image (Masson’s trichrome stain). Layered structure of the sacrotuberous ligament and obturator internus fascia. The membrane extending from the sacrotuberous ligament toward the pelvic side is notably thick, whereas the proper fascia of the obturator internus muscle is relatively thin. The obturator internus nerve runs along the muscle side of the obturator internus fascia, occupying a layer separate from that of the pudendal nerve. LA, levator ani muscle; OI, obturator internus muscle; OIN, obturator internus nerve; PA, pudendal artery; PN, pudendal nerve; Re, rectum

Coronal section examination of the right pelvis revealed that the pudendal nerve was enclosed by fascia on its medial and lateral aspects. The membrane extending from the sacrotuberous ligament toward the pelvic side was notably thick, whereas the proper fascia of the obturator internus muscle was relatively thin. The obturator internus nerve ran along the muscle side of the obturator internus fascia, occupying a layer distinct from that containing the pudendal nerve (Fig. 3).

## Discussion

### Summary of the main results

This anatomical study revealed that the pudendal nerve runs within the fascia extending from the sacrotuberous ligament and does not traverse the proper fascia of the obturator internus muscle. The proper fascia of the obturator internus muscle lies closer to the muscle, and the obturator internus nerve courses within this fascial layer. Macroscopic and histological observations confirmed that the proper fascia of the obturator internus is relatively thin, whereas the fascia derived from the sacrotuberous ligament is notably thicker.

### Results in the context of published literature

Various surgical approaches have been used to remove tumors invading the pudendal canal, and some studies recommend resection of the sacrotuberous ligament, sacrospinous ligament, and ischial spine [1, 3]. Consistent with previous findings, our results indicate that the pudendal nerve is enclosed by fascia originating from the sacrotuberous ligament.

The muscles and fasciae within the pelvis form a network of interconnected structures. Recent anatomical studies have shown that the muscles of the pelvic floor are closely linked [7, 8], with the obturator internus muscle continuing into the levator ani muscle and its associated fascia [8, 9]. Additionally, these muscles reportedly connect to the pelvic organs [7–9]. In situations where new surgical techniques have not yet become standardized, detailed anatomical investigations, such as the present study, may support preoperative anatomical understanding.

The findings of this study suggest that the pudendal nerve is enclosed by internal and external fascial layers. This dual-layered structure may result in a mismatch between pelvic and extrapelvic dissection planes. Such a mismatch can pose challenges during surgery for recurrent tumors, complicating the alignment of intraoperative dissection planes. At present, this concept is a theoretical anatomical consideration and warrants future evaluation in surgical settings.

### Strengths and weaknesses

A major strength of this study is that the dissections were performed collaboratively by anatomists and gynecologic oncologists experienced in pelvic exenteration and laterally extended endopelvic resection. In addition, the study focused on anatomical regions where dissection planes are commonly lost during surgery and highlighted differences from findings reported in previous studies.

Nevertheless, this study has certain limitations. The cadaveric specimens were obtained from individuals of advanced age (mean age > 70 years), which may have contributed to age-related muscle atrophy and limited the generalizability of the findings to younger populations. Moreover, the study was purely anatomical and did not include surgical simulations. In addition, because surgical procedures involving the pudendal canal, such as laterally extended endopelvic resection, are relatively uncommon and not yet standardized, clinical outcome data or surgical validation demonstrating the impact of these anatomical findings on oncologic or functional outcomes remain limited. Further clinical studies and cadaveric simulation research are required to establish such evidence.

### Implications for practice and future research

Future studies may extend this work to a morphological study examining the three-dimensional relationships between the sacrotuberous and sacrospinous ligaments, as well as fascial fusion patterns that are important during pelvic exenteration. Further studies incorporating cadaveric surgical models may help to further explore how these anatomical findings can be translated into surgical practice.

In conclusion, the pudendal nerve travels within the fascia derived from the sacrotuberous ligament and does not traverse the fascia of the obturator internus muscle. The proper fascia of the obturator internus muscle lies close to the muscle, and the obturator internus nerve courses between this muscle and its associated fascia.

## Supplementary Information

Below is the link to the electronic supplementary material.

Online Resource 1 Layered relationship between the sacrotuberous ligament, obturator internus muscle, pudendal nerve, and obturator internus nerve. This cadaveric video first presents a ventral (pelvic) view, identifying the obturator internus muscle, obturator internus nerve, and pudendal nerve. The specimen is then rotated to show a dorsal (perineal) view, in which the sacrotuberous ligament is clearly visualized. After reflection of the sacrotuberous ligament, the pudendal nerve is shown to course within the ligament-derived fascial layer, forming the pudendal canal. In contrast, the obturator internus nerve is observed to enter and run within a distinct layer located between the obturator internus muscle and its proper fascia. This step-by-step demonstration highlights the distinct fascial compartments of the pudendal and obturator internus nerves (WMV 126 MB)

## Data Availability

The datasets generated and/or analyzed during the current study are available from the corresponding author upon reasonable request.

## Glossary

Alcock’s canal (pudendal canal): A sheath formed by the obturator fascia that encloses the pudendal nerve and internal pudendal vessels located along the lateral wall of the ischioanal fossa.
Cadaveric dissection: A method of anatomical study involving the careful dissection of preserved human cadavers to observe and document structures.
Decalcification: A process in histology where calcium is removed from tissues (usually bone or calcified structures) to allow for easier sectioning and staining.
Fascia: Connective tissue that surrounds the muscles, nerves, and blood vessels. In this study, different layers of fascia are discussed in relation to the pelvic structures.
Histological analysis: The microscopic examination of tissue sections to study the anatomy and structure at the cellular level, often using staining techniques like Masson’s trichrome.
Ischioanal fossa: A fat-filled space located lateral to the anal canal that allows expansion during defecation and provides access to the pudendal canal.
Laterally extended endopelvic resection: An advanced surgical technique used to remove recurrent pelvic tumors, often involving wide resection of pelvic structures, including ligaments and neurovascular bundles.
Masson’s trichrome stain: A special histological staining technique used to differentiate between collagen (blue or green), muscle (red), and other tissue types.
Obturator internus muscle: A muscle located in the lateral pelvic wall that assists in lateral rotation of the thigh; it plays an important role in forming boundaries for the pudendal canal.
Obturator internus nerve: A nerve that innervates the obturator internus muscle, passing between the muscle and its proper fascia.
Pudendal nerve: A major nerve of the pelvis that supplies sensation to the external genitalia and perineum and motor function to pelvic floor muscles, i.e., the focus of this study.
Sacrotuberous ligament: A ligament extending from the sacrum to the ischial tuberosity; it contributes to the structure of the pudendal canal and is surgically relevant in pelvic resections.
Sacrotuberous ligament fascia: Connective tissue associated with the sacrotuberous ligament found to envelop the pudendal nerve in this study.
Transabdominal approach: A surgical route accessing the pelvis through the abdominal cavity, often used in laparoscopic or robotic surgery.
Transperineal approach: A surgical route accessing pelvic structures through the perineum (the area between the genitals and anus), often with the patient in the prone position.
